# Evaluation of the relationship between shear wave elastography measurements with laboratory and Doppler US parameters in patients with adult Hashimoto’s thyroiditis

**DOI:** 10.3906/sag-2008-210

**Published:** 2021-04-30

**Authors:** Nurten ANDAÇ BALTACIOĞLU, Ferhan MANTAR, Efe SOYDEMİR

**Affiliations:** 1 Department of Radiology, VKV American Hospital, İstanbul Türkiye; 2 Department of Endocrinology, VKV American Hospital, İstanbul Türkiye; 3 Department of Radiology, Ministry of Health-Marmara University Pendik Education and Research Hospital, İstanbul Türkiye

**Keywords:** Doppler, elastography, Hashimoto

## Abstract

**Background/aim:**

Hashimoto thyroiditis (HT) is an autoimmune disease that leads to tissue stiffening secondary to lymphocyte infiltration of the thyroid gland. Gray-scale ultrasound (US) is widely used in its diagnosis. Numerous studies have been conducted comparing elastography findings of HT with tissue stiffness and immunoreactivity levels. This study aims to reveal the relationship between shear wave elastography (SWE) and Doppler parameters in patients with HT.

**Materials and methods:**

The study group consisted of 45 patients diagnosed with HT, and 20 control patients without thyroid pathology. Thyroid-stimulating hormone (TSH) and antithyroid peroxidase (TPO) values were examined in patients with HT. Thyroid gland volume and echo patterns were evaluated in the gray-scale US. Doppler measurements –peak systolic velocity (Vmax), end-diastolic velocity (Vmin), pulsatility index (PI), resistivity index (RI)– from the superior thyroidal artery and SWE measurements were taken from both thyroid lobes.

**Results:**

The mean age of men and women in the HT group was 44.8 and 43.4 years. The mean TSH value (normal value: 0.3–4.2 uIU/mL) was 3.90 ± 6.6 uIU/mL, and the anti-TPO value (normal value: < 35 IU/mL) was 235.47 ± 271.12 IU/mL. The average thyroid gland volume was 10.12 ± 2.71 mL in the HT group and 6.62 ± 2.11 mL in the control group (P = 0.034). HT group mean Vmax, mean Vmin, mean PI and RI values were significantly lower compared to normal subjects (P = 0.022, P = 0.026, P = 0.042, P = 0.046, respectively). The average SWE value of the thyroid gland was 24.56 ± 18.04 kPa in the experimental group and 7.34 ± 3.54 (P < 0.05) in the control group.

**Conclusion:**

A positive correlation was found between PI and RI values and elastography values. An increase in SWE and decreases in Vmax and Vmin were found as high diagnostic value for HT.

## 1. Introduction

Hashimoto thyroiditis (chronic lymphocytic thyroiditis/chronic autoimmune thyroiditis) is one of the primary causes of hypothyroidism in children and adults [1–3]. It is common in middle-aged women [4]. Ultrasonography is very important in the diagnosis of HT, as in all thyroid gland diseases. Thyroid gland volume and echo patterns are used for diagnosis in the conventional US. In HT, pathologically, degeneration, and fibrosis develop secondary to autoimmune lymphocytic infiltration of the thyroid gland [5]. Fibrosis causes the hardening of the thyroid tissue. The stiffness of the tissue is directly related to the level of immune damage and therefore the disease [6]. 

Doppler US has been widely used in the diagnosis of thyroid diseases, especially in the differentiation of malignant and benign nodules [7,8]. However, there is not much data on diffuse thyroid diseases. Reported studies are mostly related to macroscopic blood supply patterns in color Doppler US. Doppler US also provides information about microvascular blood flow changes [9]. Spectral Doppler waveform is a result of vascular compliance and vascular resistance. Therefore, Doppler indices are affected by both vascular wall pathologies, such as atherosclerosis, and parenchymal pathologies, such as fibrosis. Resistive index (RI) and pulsatility index (PI) are the two most commonly used Doppler parameters in the evaluation of microvascular tissue blood supply. In recent years, elastography has been widely used to evaluate tissue stiffness in various pathologies [10]. There are two types of elastography: strain and shear wave elastography (SWE). Strain elastography is concerned with tissue elasticity qualitatively or semiquantitatively, but it is operator dependent. SWE, on the other hand, is a method that makes quantitative measurement independent of the performer. Recently, there have been many studies in children and adults comparing the elastography findings of the thyroid gland and its pathologies, especially HT, the degree of tissue stiffness, and immunoreactivity [6,11–14]. However, to the best of our knowledge, there is no study comparing quantitative elastography findings with Doppler parameters and evaluating the relationship between them. In this study, we aimed to evaluate the tissue stiffness with SWE and to reveal the relationship between elastography value and Doppler parameters in patients with HT.

## 2. Materials and methods

### 2.1. Patients 

The study group consists of 45 patients diagnosed with HT. Besides, 20 normal subjects without any thyroid pathology were evaluated as the control group. The diagnosis of HT was made by clinical findings, presence of thyroid autoantibodies (anti-TPO) in laboratory tests, and gray-scale US findings. Demographic (age, sex) and laboratory findings –TSH, anti-TPO– were recorded for each patient. 

Conventional/Doppler US and SWE examinations were performed using the GE Healthcare Logic E9 US device and ML 6-15 MHz linear probe. All of the examinations were conducted by a single radiologist with 25 years of experience in general US and thyroid US and 5 years of experience in elastography.

### 2.2. Gray-scale and Doppler US examination

**Figure 1:**
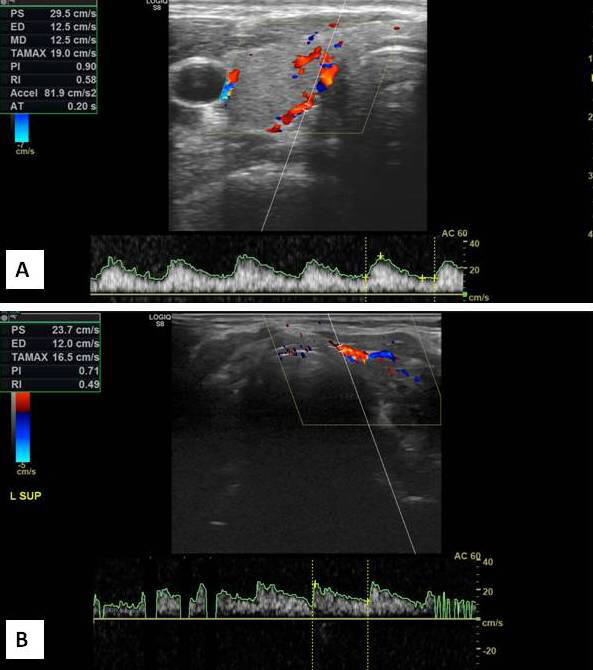
A. 55-year-old, male, normal thyroid tissue. Doppler examination of the right superior thyroidal artery. B. 56-yearold, female, Hashimoto’s thyroiditis. Doppler examination of the left superior thyroidal artery. Compared with normal tissue, PI and RI values and Vmax and Vmin decreased in accordance with the increased tissue resistance (Doppler indices are on the images).

Conventional US examination was performed with the patient in the supine position and head extension. Three dimensions –depth (d), width (w), length (l)– of both thyroid lobes and isthmus thickness were measured. The volume of each lobe was calculated by the formula: V (mL) = d × w × l × 0.52 cm. The thyroid volume is the sum of the volumes of both lobes. The volume of the isthmus is not included. The echo pattern of the thyroid parenchyma was evaluated. US findings were staged according to the evaluation system defined by Sostre and Reyes [15]. Later, Doppler indices –Vmax, Vmin, PI, RI– were measured from the level where the superior thyroidal arteries entered the thyroid parenchyma or the descending tract of the artery (Figures 1A and 1B). The Doppler angle was always set at 60°. To detect low flow, pulse repetition frequency was set as low as possible, or the color gain was set high and then adjusted until background noise just disappears. 

### 2.3. Shear wave elastography examination

**Figure 2:**
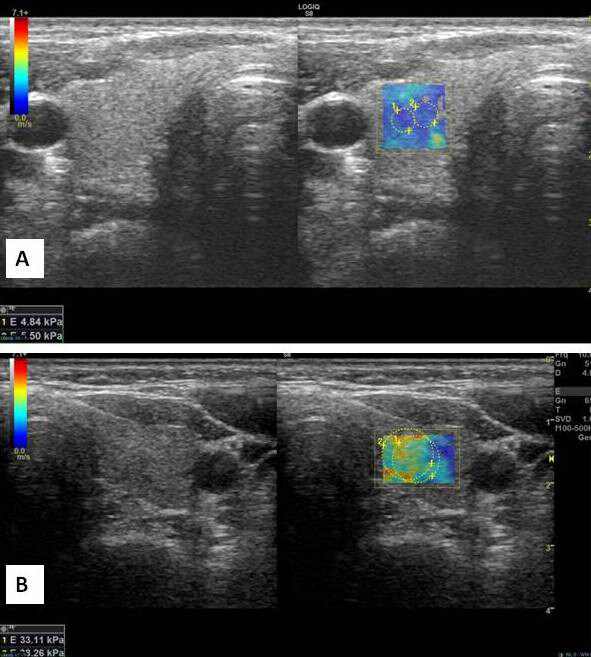
SWE examinations of the patients in Figure 1. A. Low elastography values (4.84 and 5.50 kPa) consistent with normal thyroid tissue, and B. high values (33.11 and 38.26 kPa) consistent with increased tissue hardness were measured.

SWE measurements were made using 10 mm sized ROIs from two or three separate points of approximately similar depth in both thyroid lobes. Vascular tissues within the measurement area were avoided. Measurements were recorded in kilopascals (kPa). ROIs were located in the regions with the highest degree of hardness in elasticity mapping. The average hardness level was calculated for each lobe by taking the average of the measurements (Figures 2A and 2B). 

### 2.4. Statistical analysis

Besides descriptive statistics, the Mann–Whitney U test was used to compare continuous data between groups. Pearson’s correlation coefficient was used to measure the association between continuous variables. To show the efficiency of SWE values and Doppler parameters in the diagnosis of HT, receiver operating characteristic (ROC) analysis was used. The results were evaluated at a 95% confidence interval. P < 0.05 was considered significant.

## 3. Results

Demographic characteristics and laboratory findings of the patient group are given in Table 1, and the gray-scale US, Doppler US, and SWE findings are given in Table 2. Eleven of the patients were male and 34 were female. The average age was 44.8 (20–63) years for males and 43.4 (23–67) years for females. The control group consisted of 5 men and 15 women. The average age was 35.6 (29–44) years.

**Table 1:** Demographics, laboratory findings.

	Patients (n = 45)	Control (n = 20)
Age (mean) (min-max)Male Female	50.6 (22–71)44.8 (22–63)43.4 (23–71)	35.6 (29–44)36.4 (32–41)35.3 (29–44)
Sex, Male/Female	11/34	5/15
TSH (0.3–4.2 uIU/mL)	3.90 ± 6.6	-
anti-TPO (< 35 IU/mL)	235.47 ± 271.12	-

TSH: thyroid stimulating hormone, TPO: thyroid peroxidase.

**Table 2:** Conventional US, Doppler US, elastography findings.

	Patients (n = 45)			Control (n = 20)			P‡
Gray-scale US (Sostre classification)Grade IGrade IIGrade IIIGrade IV	141894			*			
Thyroid volume (mL)Right lobLeft lobTotal	5.49 ± 3.514.63 ± 2.61	P‡		3.15 ± 1.173.47 ± 1.34	P‡		0.034
		0.205			0.428		
	10.12 ± 2.71			6.62 ± 2.11			
Vmax (mean, cm/s)	27.78			38.22			0.022
Vmin (mean, cm/s)	12.01			16.93			0.026
PI	0.72			0.84			0.042
RI	0.49			0.56			0.046
SWE (mean, kPa)Right lobLeft lobMean	24.34 ± 18.9724.77 ± 17.12		P‡	6.46 ± 3.328.22 ± 3.76		P‡	0.0132
			0.294			0.309	
	24.56 ± 18.04			7.34 ± 3.54			

*Thyroid US examinations are completely normal, PI: pulsatility index, RI: resistivity index, SWE: shear wave elastography, kPa: kilo Pascal, ‡ Mann–Whitney U test.

### 3.1. Laboratory findings

For the HT patient group, the mean TSH value (normal value: 0.3–4.2 uIU/mL), and the mean anti-TPO value (normal value: < 35 IU/mL) were as 3.90 ± 6.6 uIU/mL, and 235.47 ± 271.12 IU/mL respectively.

### 3.2. Gray-scale and Doppler US examination

In the conventional US assessment, according to the Sostre and Reyes classification; 14 patients were grade I, 18 were grade II, 9 were grade III, and 4 were grade IV. The mean volume of the thyroid gland was 10.12 ± 2.71 mL for the HT group, and 6.62 ± 2.11 mL for the control group. For both groups, there was no significant difference between right and left thyroid lobes. In patients with HT, thyroid gland volumes were significantly larger than those of the members in the healthy group (P = 0.034).

Average systolic-peak velocities were 27.78 cm/s in HT group and 38.22 cm/s in control group (P = 0.022), average end-diastolic velocities were 12.01 cm/s in HT group and 16.93 cm/s in control group (P = 0.026), average PI values were 0.72 in HT group and 0.84 in control group (P = 0.042), and average RI values were 0.49 in HT group and 0.56 (P = 0.046) in control group.

### 3.3. Shear wave elastography examination

Correlation analysis between the laboratory values, elastography measurements, and the Doppler indices are given in Table 3. Pearson’s correlation coefficient was calculated as r = 0.022 between SWE value and TSH value, indicating a weak positive relationship. A significant positive correlation was observed between SWE value and anti-TPO value (Pearson’s correlation coefficient r = 0.511).

**Table 3:** Correlation analysis between the laboratory values, elastography measurements, and the Doppler indices.

	Patients (n = 45)		Control (n = 20)	
SWETSHanti-TPO	r	P‡		
	0.022 0.511	0.886 0.001		
SWEVmaxVminPIRI	r	P‡	r	P‡
	–0.2968–0.23670.60170.6081	0.0480.1170.0000.000	–0.1563–0.3117 0.54610.6205	0.5110.1810.0120.003

r: Pearson’s correlation coefficient.

The mean SWE value measured in the patient group was 24.34 ± 18.97 kPa for the right thyroid lobe and was 24.77 ± 17.12 kPa for the left lobe. There was no significant difference between right and left thyroid lobes (P = 0.294). In the control group, the mean SWE values measured for the right and the left lobes were 6.46 ± 3.32 kPa and 8.22 ± 3.76 kPa respectively, and no significant difference was detected as well (P = 0.309). The mean SWE value for the whole thyroid gland was 24.56 ± 18.04 kPa in the patient group and 7.34 ± 3.54 in the control group. There was a significant difference between the two groups (P = 0.0132).

**Figure 3:**
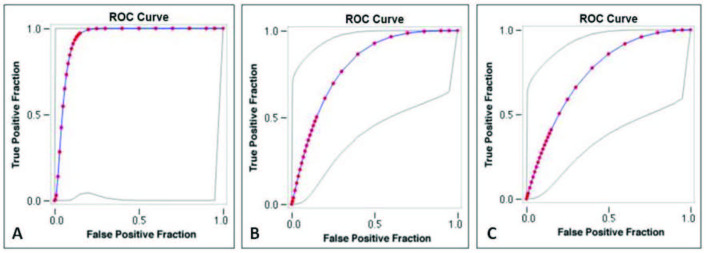
ROC analysis: Increase in SWE (A), decrease in systolic-peak velocity (B) and decrease in end-diastolic velocity (C) show high diagnostic value for Hashimoto thyroiditis (AUC: 0.945, 0.801, and 0.747, respectively).

**Figure 4:**
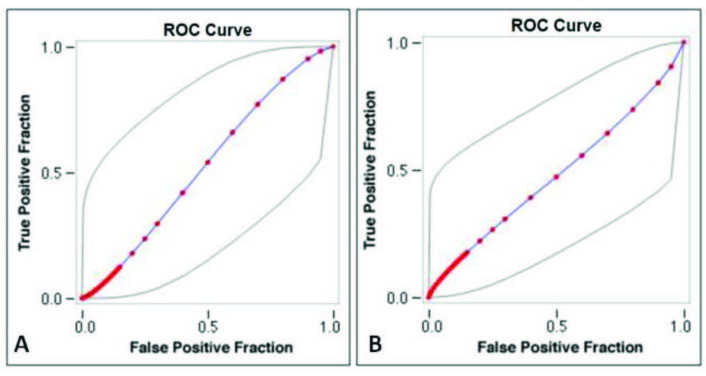
ROC analysis: The distinctive feature of PI (A) and RI (B) in terms of Hashimoto’s thyroiditis is poorly observed (AUC: 0.525 and 0.429, respectively).

In evaluating the relationship between SWE values and Doppler parameters; weak negative correlation with systolic-peak velocity and end-diastolic velocity (Pearson’s correlation coefficient –0.2968 and –0.2367, respectively), significant positive correlation with PI and RI values (Pearson’s correlation coefficient 0.6017 and 0.6081, respectively) were determined. In ROC analyzes, it was observed that the diagnostic value of the increase in SWE and the decrease in systolic-peak velocity and end-diastolic velocity were high (AUC: 0.945, 0.801 and 0.747, respectively) (Figures 3A–3C). Although there is a positive correlation between PI, and RI values and SWE, the distinguishing feature of PI and RI in ROC analyzes was calculated to be quite weak (AUC: 0.525 and 0.429, respectively) (Figures 4A and 4B).

## 4. Discussion

Many studies have been conducted on the use of gray-scale US, Doppler US, and US elastography in the diagnosis and differentiation of thyroid gland pathologies in adult and pediatric age groups. The conventional US and Doppler US examinations have high sensitivity in detecting pathologies of superficial organs such as the thyroid. Evaluation of blood supply patterns of tissues or lesions in color Doppler US is widely used for differential diagnosis. However, their specificity is low in both focal and diffuse thyroid diseases [16,17]. Although risk assessment systems, such as thyroid imaging reporting and data system (TI-RADS), scoring malignancy probability or pattern-based diagnostic systems as in the American Thyroid Association (ATA) guidelines have been developed for the differential diagnosis of benign and malignant thyroid nodules, fine needle aspiration biopsy is still used as the most important tool in diagnosis [18,19].

Showing the blood supply pattern with Doppler US provides information about the macrovascular circulation of the lesion or organ. Although increased blood supply is defined as pathognomonic for Graves’ disease with hyperthyroidism, increased vascularity can also be observed in HT [20]. Therefore, studies on the use of Doppler parameters have been carried out, especially in the differential diagnosis of diffuse thyroid diseases, due to their possible additional contributions. When compared with HT, it has been shown that intraparenchymal systolic-peak velocities increase significantly in Graves’ disease [21]. Caruso et al. stated that, while the systolic-peak velocities measured from the inferior thyroidal artery in Graves’ disease were above 150 cm/s, they reported that the peak velocities were within normal limits (< 65 cm/s) in other common thyroid gland diseases including HT [22]. In our patient group, mean systolic-peak velocities were measured as 27.78 cm/s (17.8–56.2 cm/s) in HT group and 38.22 cm/s (24.8–58 cm/s) in control group in accordance with this study. In a study evaluating two groups of children with normal children and children with endemic goiter, the mean RI value was found to be 0.58 and 0.70, respectively [23]. In another study, patients with HT were compared in terms of gray-scale US patterns and RI values, and no significant difference was found between patients with widespread changes in the US and patients with minimal parenchymal changes (0.56 and 0.58, respectively) [24]. Apart from this, only one study was found to compare the multiparametric (RI, SWE, acceleration time) Doppler US findings of five different groups, including a normal control group, patients with HT, and multinodular hyperplasia [25]. The mean RI of the HT group was 0.49 and the mean RI of the normal control group was 0.56, and the difference was considered significant.

Although both RI and PI are used to assess the resistance to flow in the vascular system, RI is actually an index of pulsatility [26]. Tissue vascular resistance is a hemodynamic parameter that has the least influence on RI, compared with vascular compliance, systemic pulse pressure, and heart rate and rhythm [27]. PI, on the other hand, is typically used to assess the resistance in a pulsatile vascular system. That is why, in our study, we evaluated not only RI but also PI, Vmax, and Vmin parameters. In patients with HT, mean Vmax, Vmin, average PI, and average RI values were significantly lower than the control group due to high vascular resistance in accordance with the increase in the degree of stiffness of the tissue.

Many studies on the diagnostic value of SWE and the relationship between the elastography value of the tissue and the level of thyroid autoantibodies have been reported in patients with HT [6,12,28]. In general, as in our study, SWE values were found to be higher in patients with HT compared to normal patients. Similarly, thyroid autoantibody levels show a positive correlation with elastography values. In our patient group, while there was a weak correlation between SWE and TSH values, a positive correlation was observed between SWE and anti-TPO values.

Fibrosis and hardening of the thyroid tissue due to lymphocyte infiltration in HT is the underlying pathological reason for the findings and quantitative values obtained in the multiparametric US examination. As a result, while the hardening in the tissue increases the SWE value, it is expected that there will be changes in Doppler parameters due to increased tissue resistance and vascular resistance. The most important feature of this study that distinguishes it from previously published studies is the investigation of a possible relationship between different physical characteristics (SWE vs. Vmax, Vmin, PI, RI) that are affected on the same parenchymal pathology background. A weak negative correlation was found between SWE and systolic-peak velocity and end-diastolic velocity, and a strong positive correlation was found between PI and RI values. In ROC analyzes, it was calculated that the diagnostic value of the increase in SWE and the decrease in systolic-peak velocity and end-diastolic velocity in terms of HT was high, and the distinguishing feature of PI and RI was quite weak.

The most important limitation of the study is that all examinations are performed by a single operator. In order to minimize possible subjective errors in a significantly operator-dependent modality such as the US, the simultaneous evaluation of the entire study group by two different practitioners would also be effective in revealing the differences between practitioners.

In conclusion, the multiparametric US examination is very valuable in evaluating the pathologies of superficial organs such as the thyroid. In addition to the gray-scale US, Doppler US has been widely used for this purpose for a long time. Elastography is another relatively new and less used US method in which the hardness of the tissue is evaluated. Since both Doppler parameters and elastography values are a reflection of the physical properties of the tissues, a positive correlation was found especially between PI and RI values and elastography. However, in order to create a mathematical correlation between Doppler indices and elastography values –to use them interchangeably– it is necessary to conduct studies in larger patient and control groups.

## Informed consent

Informed consent form was obtained from the patients for US examinations. Approval for the study was obtained from the Ethics Committee of Marmara University Faculty of Medicine with the decision dated 08.08.2020 and numbered 09.2020.866.
